# NOP2/Sun RNA methyltransferase 2 is a potential pan-cancer prognostic biomarker and is related to immunity

**DOI:** 10.1371/journal.pone.0292212

**Published:** 2023-09-28

**Authors:** Lemei Zheng, Mengna Li, Jianxia Wei, Shipeng Chen, Changning Xue, Yumei Duan, Faqing Tang, Guiyuan Li, Wei Xiong, Kelin She, Hongyu Deng, Ming Zhou

**Affiliations:** 1 NHC Key Laboratory of Carcinogenesis, Hunan Key Laboratory of Oncotarget Gene, Hunan Cancer Hospital and the Affiliated Cancer Hospital of Xiangya School of Medicine, Central South University, Changsha, China; 2 Cancer Research Institute, School of Basic Medical Sciences, Central South University, Changsha, China; 3 The Key Laboratory of Carcinogenesis and Cancer Invasion of the Chinese Ministry of Education, Central South University, Changsha, China; 4 Department of Thoracic Surgery, Hunan Provincial People’s Hospital, The First Affiliated Hospital of Hunan Normal University, Changsha, China; Xiangya Hospital Central South University, CHINA

## Abstract

**Background:**

NOP2/Sun RNA methyltransferase 2 (NSUN2), an important methyltransferase of m5C, has been poorly studied in cancers, and the relationship between NSUN2 and immunity remains largely unclear. Therefore, the purpose of this study was to explore the expression and prognostic value of NSUN2 and the role of NSUN2 in immunity in cancers.

**Methods:**

The TIMER, CPTAC and other databases were used to analyze the expression of NSUN2, its correlation with clinical stage and its prognostic value across cancers. Moreover, the TISIDB, TIMER2.0 and Sangerbox platform were used to depict the relationships between NSUN2 and immune molecular subtypes, tumor-infiltrating lymphocytes (TILs), immune checkpoints (ICPs) and immunoregulatory genes. Furthermore, the NSUN2-interacting proteins and related genes as well as the coexpression networks of NSUN2 in LIHC, LUAD and HNSC were explored with the STRING, DAVID, GEPIA2 and LinkedOmics databases. Finally, the subcellular location and function of NSUN2 in HepG2, A549 and 5-8F cells were investigated by performing immunofluorescence, CCK-8 and wound healing assays.

**Results:**

Overall, NSUN2 was highly expressed and related to a poor prognosis in most types of cancers and was also significantly associated with immune molecular subtypes in some cancer types. Furthermore, NSUN2 was significantly associated with the levels of ICPs and immunoregulatory genes. In addition, NSUN2 was found to be involved in a series of immune-related biological processes, such as the humoral immune response in LIHC and LUAD and T-cell activation and B-cell activation in HNSC. Immunofluorescence and CCK-8 assays also confirmed that NSUN2 was widely expressed in the nucleus and cytoplasm, and overexpression of NSUN2 promoted the proliferation and migration of HepG2, A549 and 5-8F cells. NSUN2 was also confirmed to positively regulate the expression of PD-L1.

**Conclusion:**

NSUN2 serves as a pan-cancer prognostic biomarker and is correlated with the immune infiltration of tumors.

## Introduction

The crucial role of RNA epigenetic modifications in tumors has gained increasing attention in recent years, of which the most studied modifications are N6-methyladenosine (m6A), 5-methylcytosine (m5C) and N1-methyladenosine (m1A) [1–3]. M5C, a kind of posttranscriptional RNA modification [4], is generally considered to be related to the modification of transfer RNAs (tRNAs) and ribosomal RNAs (rRNAs) [5, 6]. However, mounting evidence attests that in addition to rRNA and tRNA, m5C modification is also involved in the regulation of messenger RNA (mRNA) and other types of RNA [7, 8]. Some studies have demonstrated that m5C modification participates in tumorigenesis and tumor progression. For example, m5C reader Y-box binding protein 1 can recruit ELAV-like RNA binding protein 1 (ELAVL1) in bladder cancer (BLCA), thus increasing the stability of hepatoma-derived growth factor (HDGF) mRNA and promoting the invasion of BLCA cells [9]. In addition, the regulatory factor of m5C modification is related to the prognosis of pancreatic ductal adenocarcinoma (PDAC) and head and neck squamous cell carcinoma (HNSC) [10, 11], which suggests that the molecules that regulate m5C modification might play critical roles in tumor progression.

NOP2/Sun RNA methyltransferase 2 (NSUN2), a member of the NOL1/NOP2/SUN domain (NSUN) family, is an important methyltransferase of m5C modification. Studies have proven that NSUN2 is crucial in tumorigenesis and malignant progression [12]. For example, the m5C modification mediated by NSUN2 promotes poor differentiation in hepatocellular carcinoma (HCC) [13]. Meanwhile, NSUN2 promotes not only the proliferation and metastasis of gastric cancer (GC) cells [14] but also the occurrence and development of esophageal squamous cell carcinoma (ESCC) and gallbladder carcinoma (GBC) [15, 16]. These results reveal that NSUN2 is associated with carcinogenesis and tumor progression.

Although NSUN2 has been confirmed to exert critical roles in m5C modification and tumor progression in some types of cancers, NSUN2 has not been studied systematically across cancers, and the mRNA and protein expression of NSUN2, the prognostic value of NSUN2 and the relationship between NSUN2 and immunity remain largely unclear. In this study, we investigated the expression profiles of NSUN2 in multiple tumor types and demonstrated the relationship between NSUN2 and tumor prognosis and immunity, which explores the potential of NSUN2 in immunotherapy and provides a promising strategy for the clinical diagnosis and treatment of tumors.

## Materials and methods

### Analysis of NSUN2 expression across cancers

The mRNA expression of NSUN2 was investigated with the TIMER database (http://timer.cistrome.org/) in different cancer tissues and paired adjacent normal tissues [17]. TCGA/TARGET/GTEx (PANCAN, N = 19131, G = 60499), a pan-cancer dataset, was downloaded from UCSC (https://xenabrowser.net/) websites [18, 19]. The expression value of the NSUN2 gene from each sample was extracted and further transformed with a log2(x+0.001) transformation in the Sangerbox platform [20, 21]. In addition, the open access proteomic online tool CPTAC (http://ualcan.path.uab.edu/analysis-prot.html) was used to describe the protein expression of NSUN2 in different types of tumors, and representative immunohistochemical images of NSUN2 in 8 normal tissues and 8 tumor tissues were obtained from the HPA web portal (http://www.proteinatlas.org/).

### The clinical correlation analysis of NSUN2

The cancer stage expression of NSUN2 in multiple tumor types was depicted by the UALCAN website (http://ualcan.path.uab.edu), which is based on TCGA data [22]. The Kaplan‒Meier plotter (http://kmplot.com/analysis/) database and PrognoScan (http://dna00.bio.kyutech.ac.jp/PrognoScan/ index) website that uses GEO datasets were utilized to calculate the overall survival (OS) and relapse-free survival (RFS) data of NSUN2 in different tumor types [23, 24]. When the *P* value was less than 0.05, the difference was considered to be statistically significant.

### RNA modification analysis of NSUN2

The expression data of NSUN2 and 44 genes related to RNA modification were extracted from the downloaded pan-cancer dataset TCGA/TARGET/GTEx and visualized by the Sangerbox platform.

### Subtype and immune analysis based on NSUN2 expression

The expression of NSUN2 in different cell types was explored with the TISCH database (http://tisch.comp-genomics.org/documentation/), GeneCards (https://www.genecards.org/) web portal and HPA website [25]. The relationships between NSUN2 and immune subtypes and molecular subtypes were described by the TISIDB database [26]. In addition, the R software package IOBR with the TIMER method and the psych package within the Sangerbox platform were used for the correlation analysis between NSUN2 expression and the levels of B cells, CD4 T cells, CD8 T cells, neutrophils, macrophages and dendritic cells (DCs) [20]. Moreover, the correlations between NSUN2 and ICPs and immunoregulatory genes were further analyzed. In addition, the relationship between NSUN2 and immunity was verified and analyzed via the TIMER website. *P* < 0.05 indicates a significant difference.

### Analysis of NSUN2-related genes

Twenty-two proteins interacting with NSUN2 were obtained from the STRING websites (https://string-db.org/). The number of proteins was set to not exceed 50, active interaction sources were selected as “experiments”, and medium confidence was set to 0.4. Subsequently, the top 150 genes most related to NSUN2 were obtained via GEPIA2 (http://gepia2.cancer-pku.cn/#analysis), and the associations between NSUN2 and the top five genes in 33 tumor types were further analyzed by the TIMER database. Finally, the obtained proteins and 150 genes were subjected to GO and KEGG enrichment analysis via the DAVID web portal and the Sangerbox platform [27].

### Coexpression network analysis of NSUN2 in LIHC, LUAD and HNSC

Upregulated and downregulated genes in the RNA expression data of LIHC, LUAD and HNSC were explored with the LinkedOmics database, and the enrichment of NSUN2-related genes was further studied (set at FDR<0.05) [28]. *P* < 0.05 was considered to indicate statistical significance.

### Immunofluorescence analysis

Three representative cell lines, HepG2 (LIHC), A549 (LUAD) and 5-8F (nasopharyngeal carcinoma), were seeded on the coverslips and cultured at 37°C for 12 h. After fixation for 15 min with 4% formaldehyde, the cells were permeabilized with 0.3% Triton X-100. Then, the three cell lines were incubated with anti-NSUN2 antibody at 4°C for 14 h. After that, Alexa Fluor 488-conjugated donkey anti-rabbit IgG (H+L) was added to the coverslip and incubated at 37°C for 1 h. Finally, the coverslip was stained with DAPI and subsequently photographed with a fluorescence microscope (Olympus, Tokyo, Japan).

### Western blot analysis

Cells were lysed with lysis buffer (NCM Biotech, Suzhou, China) supplemented with cocktail. A total of 30–40 μg of protein was separated by 10% SDS-polyacrylamide gel electrophoresis, and the PVDF membranes (Millipore, Billerica, USA) were blocked with 5% nonfat milk. Then, the membranes were incubated with the following primary antibodies overnight at 4°C: NSUN2 (1:1000) and GAPDH (1:10000) (Proteintech Group, Inc., Wuhan, China), and with secondary antibody for 1 h at 37°C. Finally, bands on immunoblots were detected with western blotting substrate (Share-Bio, Shanghai, China).

### CCK-8 (Cell Counting Kit-8) experiment

HepG2, A549 and 5-8F cells were seeded on 96-well plates at 1000 or 1500 cells/well. Then, the OD value was detected by adding 10 μL CCK-8 reagent (Selleck, Houston, TX, USA) and incubating for 2 h at 37°C. Finally, the optical density value was measured at 450 nm with a microplate reader (Beckman, Brea, CA, USA).

### Wound healing assay

HepG2, A549 and 5-8F cell lines were transfected for 24 h, and dashes were made in six-well plates, followed by incubation with 2% serum and imaging.

### Statistical analysis

ImageJ software was used to calculate the migration distance. GraphPad Prism 8.0 software (GraphPad, Inc., USA) was used for data analysis and figure generation. The difference between the two groups was analyzed by Student’s t test. The data in the figures represent the mean ± standard deviation (mean±SD). *P*<0.05 was considered to indicate statistical significance (* *P*<0.05, ** *P* <0.01, *** *P* <0.001).

## Results

### NSUN2 is significantly upregulated in multiple tumors

**Fig 1:**
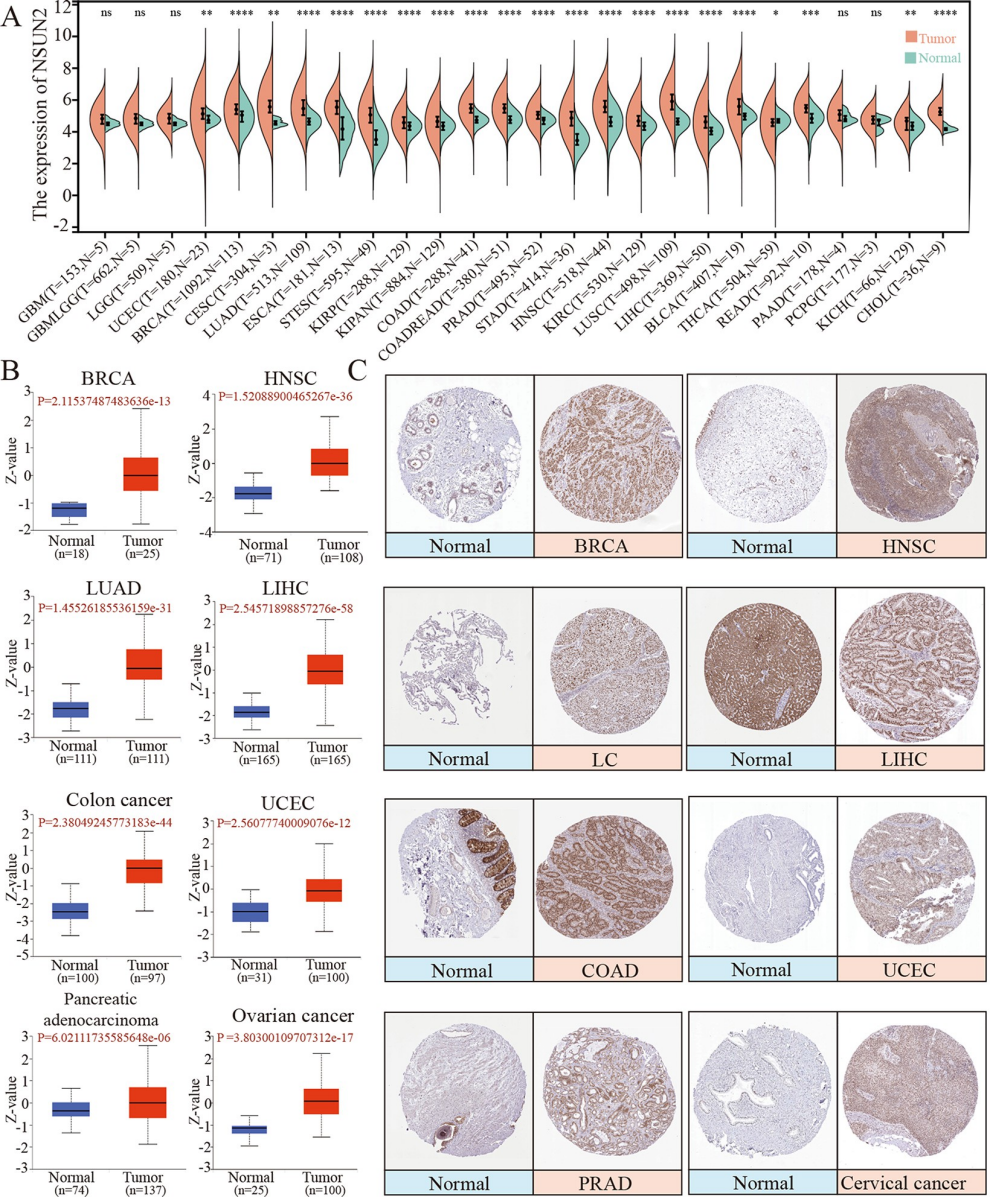
Expression of NSUN2 in different tumor types. (A) The mRNA expression of NSUN2 in some tumor types and normal tissues. T, tumor; N, normal. (B) NSUN2 protein expression in BRCA, HNSC, LUAD, LIHC, colon cancer, UCEC, pancreatic adenocarcinoma and ovarian cancer (OV). (C) IHC images of NSUN2 in BRCA, HNSC, lung cancer (LC), LIHC, COAD, UCEC, PRAD and cervical cancer (CC) (*P<0.05, **P<0.01, ***P<0.001, ****P<0.0001, ns, no significance).

The expression of NSUN2 in the RNA-seq data of TCGA was investigated with TIMER. The data showed that the mRNA level of NSUN2 was greatly upregulated in 18 of 33 tumor types compared with paired adjacent normal tissues (S1A Fig). Moreover, we evaluated the differential expression of NSUN2 between unpaired normal samples and tumor samples using the Sangerbox platform and found that NSUN2 was highly expressed in 20 of 26 tumor types (Fig 1A), including colon adenocarcinoma (COAD), kidney chromophobe (KICH), head and neck squamous cell carcinoma (HNSC), breast invasive carcinoma (BRCA), rectum adenocarcinoma (READ), lung adenocarcinoma (LUAD), liver hepatocellular carcinoma (LIHC), kidney renal clear cell carcinoma (KIRC), esophageal carcinoma (ESCA), prostate adenocarcinoma (PRAD), uterine corpus endometrial carcinoma (UCEC), stomach and esophageal carcinoma (STES), bladder urothelial carcinoma (BLCA), cervical squamous cell carcinoma and endocervical adenocarcinoma (CESC), colon adenocarcinoma/rectum adenocarcinoma esophageal carcinoma (COADREAD), pan-kidney cohort (KICH+KIRC+KIRP) (KIPAN), stomach adenocarcinoma (STAD), lung squamous cell carcinoma (LUSC), thyroid carcinoma (THCA), cholangiocarcinoma (CHOL) and kidney renal papillary cell carcinoma (KIRP). The above results proved that the mRNA level of NSUN2 is significantly increased in most tumor types.

To assess the functional products of genes (proteins) in disease, the protein expression of NSUN2 was assessed in a total of 13 tumor types via the CPTAC proteomics database. The protein level of NSUN2 was greatly increased in LUAD, LIHC, COAD, BRCA, HNSC, UCEC, pancreatic adenocarcinoma, ovarian cancer (OV), glioblastoma multiforme (GBM) and KIRC tissues vs. corresponding normal tissues (Fig 1B, S1B, S1C Fig). Moreover, we analyzed the expression of NSUN2 in the pathological tissues of different tumor types using the immunohistochemistry (IHC) images provided by the HPA database. The protein level of NSUN2 was high in a series of tumor types, such as BRCA, HNSC, lung cancer (LC), LIHC, COAD, UCEC, PRAD and cervical cancer (CC), while the expression of NSUN2 was not detected or moderate in normal clinical samples (Fig 1C). The results revealed that the mRNA and protein expression levels of NSUN2 are increased in multiple tumor types.

### Clinical correlations and prognostic value of NSUN2 in tumors

The correlation between NSUN2 expression and clinicopathological stage in different tumor types was depicted by using the UALCAN database. NSUN2 was found to be highly expressed in most tumor types compared with normal tissues and presented an increasing expression trend with increasing tumor stage in some tumor types. Taking UCEC as an example, the differential expression of NSUN2 was observed in different stages, such as stage 1 vs. stage 3 and stage 1 vs. stage 4. Moreover, the expression of NSUN2 was also different in different KIRC in stages (S2 Fig).

**Fig 2:**
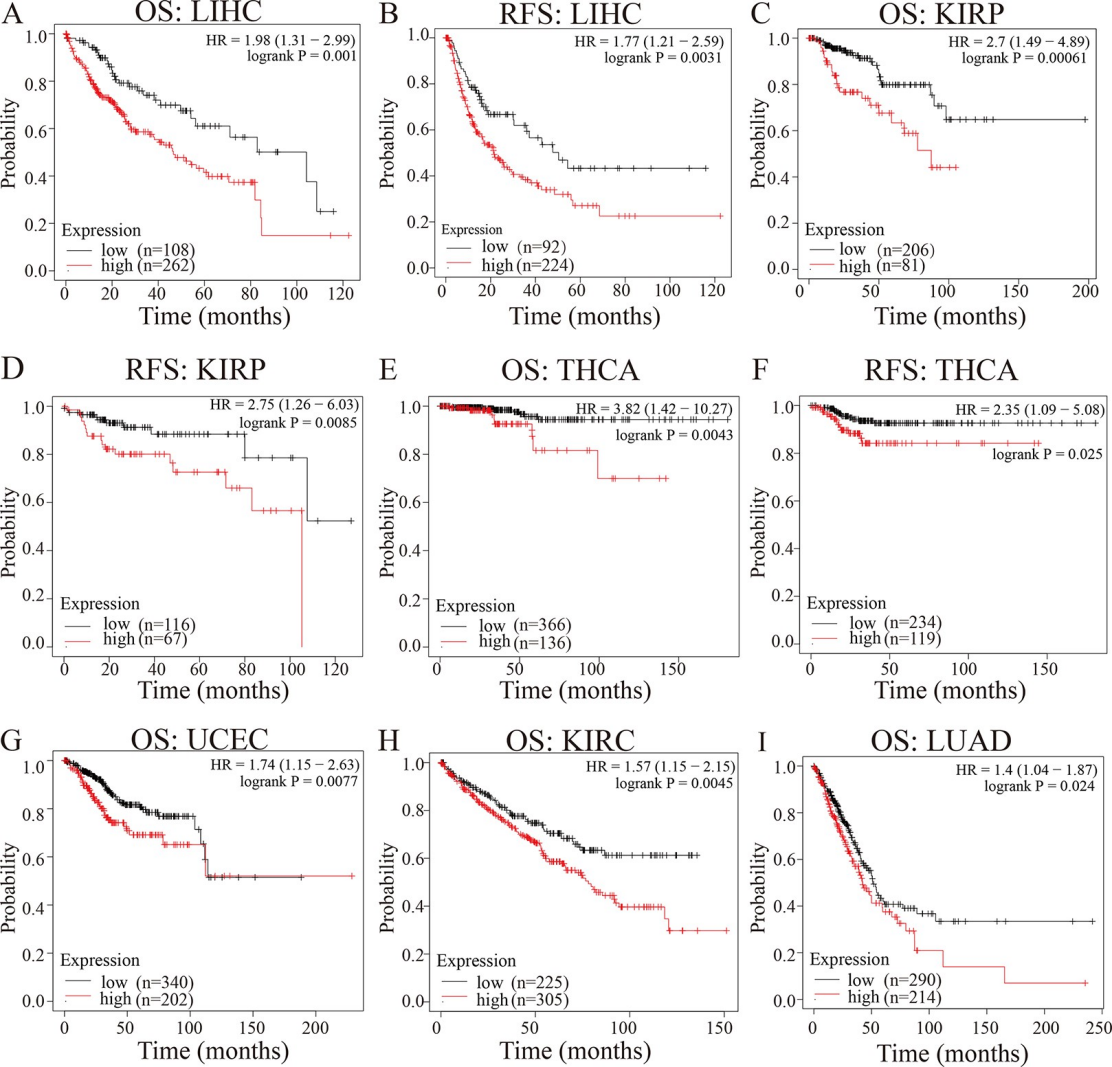
The survival curve of NSUN2 by the Kaplan‒Meier plotter database. (A) OS in LIHC cohorts. (B) RFS in LIHC cohorts. (C) OS in KIRP cohorts. (D) RFS in KIRP cohorts. (E) OS in THCA cohorts. (F) RFS in THCA cohorts. (G) OS in UCEC cohorts. (H) OS in KIRC cohorts. (I) OS in LUAD cohorts. OS, overall survival. RFS, relapse-free survival. Red line, high expression of NSUN2. Black line, low expression of NSUN2.

Subsequently, the prognostic value of NSUN2 in terms of OS and RFS was calculated using Kaplan‒Meier Plotter and PrognoScan web plotter. We found that high expression of NSUN2 can serve as an indicator of poor OS and RFS in LIHC (Fig 2A, 2B), KIRP (Fig 2C, 2D) and THCA (Fig 2E, 2F). In addition, high expression of NSUN2 indicated poor OS in UCEC (Fig 2G), KIRC (Fig 2H) and LUAD (Fig 2I). Meanwhile, high expression of NSUN2 was a risk factor in BRCA and OV according to data from the PrognoScan database (S3G–S3J Fig). More information about the prognosis of NSUN2 was shown in S3 Fig. The results revealed that the high expression of NSUN2 is associated with the prognosis of different tumor types and pathological stages of tumors, suggesting that NSUN2 might serve as a poor prognostic factor in cancers.

### RNA modification of NSUN2 across cancers

We further described the relationship between the expression of NSUN2 and RNA modification owing to NSUN2 serving as an m5C methyltransferase, including the correlation between NSUN2 and m1A, m5C and m6A modification-related regulators, including writers, erasers and readers, such as the m1A readers YTHDF1/2/3 and YTHDC1 and the m6A writers METTL3, METTL14 and WTAP. We found that NSUN2 expression was positively correlated with the expression of RNA modification-related genes in most tumor types (S4 Fig). For example, NSUN2 had a significant association with RNA modification-related genes in UCEC (S4A Fig). The results suggested that NSUN2, working as a methyltransferase of m5C, may act on the expression of downstream target genes together with other modified regulators, thus playing critical roles in tumor progression.

### The expression of NSUN2 is correlated with immune and molecular subtypes

**Fig 3:**
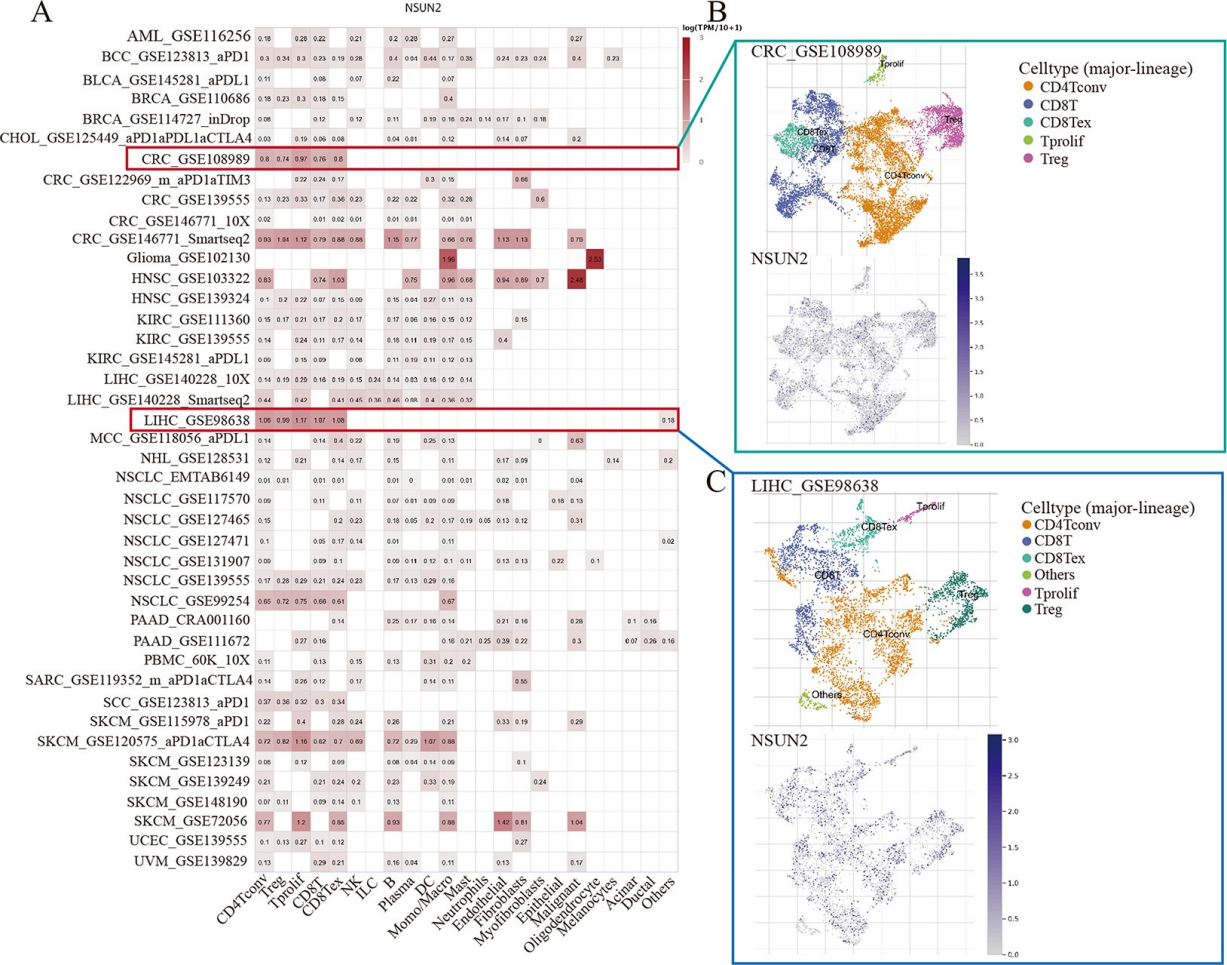
Single-cell analysis of NSUN2. (A) Expression of NSUN2 in malignant cells, stromal cells, immune cells and functional cells. (B) Scatter plot describing the expression level of NSUN2 in the GSE108989 dataset. (C) Scatter plot describing the expression level of NSUN2 in the GSE98638 dataset.

To investigate which cell type exhibits the highest expression of NSUN2, the expression of NSUN2 was explored in tumor microenvironment cells using the TISCH web tool. We found that among malignant cells, stromal cells, immune cells and functional cells, NSUN2 was relatively highly expressed in immune cells, such as CD4Tconv, monocytes/macrophages and B cells (Fig 3A). Specifically, NSUN2 was extensively expressed in immune cells such as CD4Tconv and Treg cells in the CRC microenvironment in the GSE108989 dataset (Fig 3B). In the GSE98638 of LIHC, NSUN2 was also widely expressed in immune cells such as CD4Tconv cells (Fig 3C). The protein expression of NSUN2 in immune cells was also relatively higher compared with other cell types (S4B Fig), and NSUN2 was highly expressed in most immune cells, such as Naive CD4+ T cells, but low in neutrophils (S4C Fig). These results suggest that NSUN2 might be involved in the process of immune regulation.

**Fig 4:**
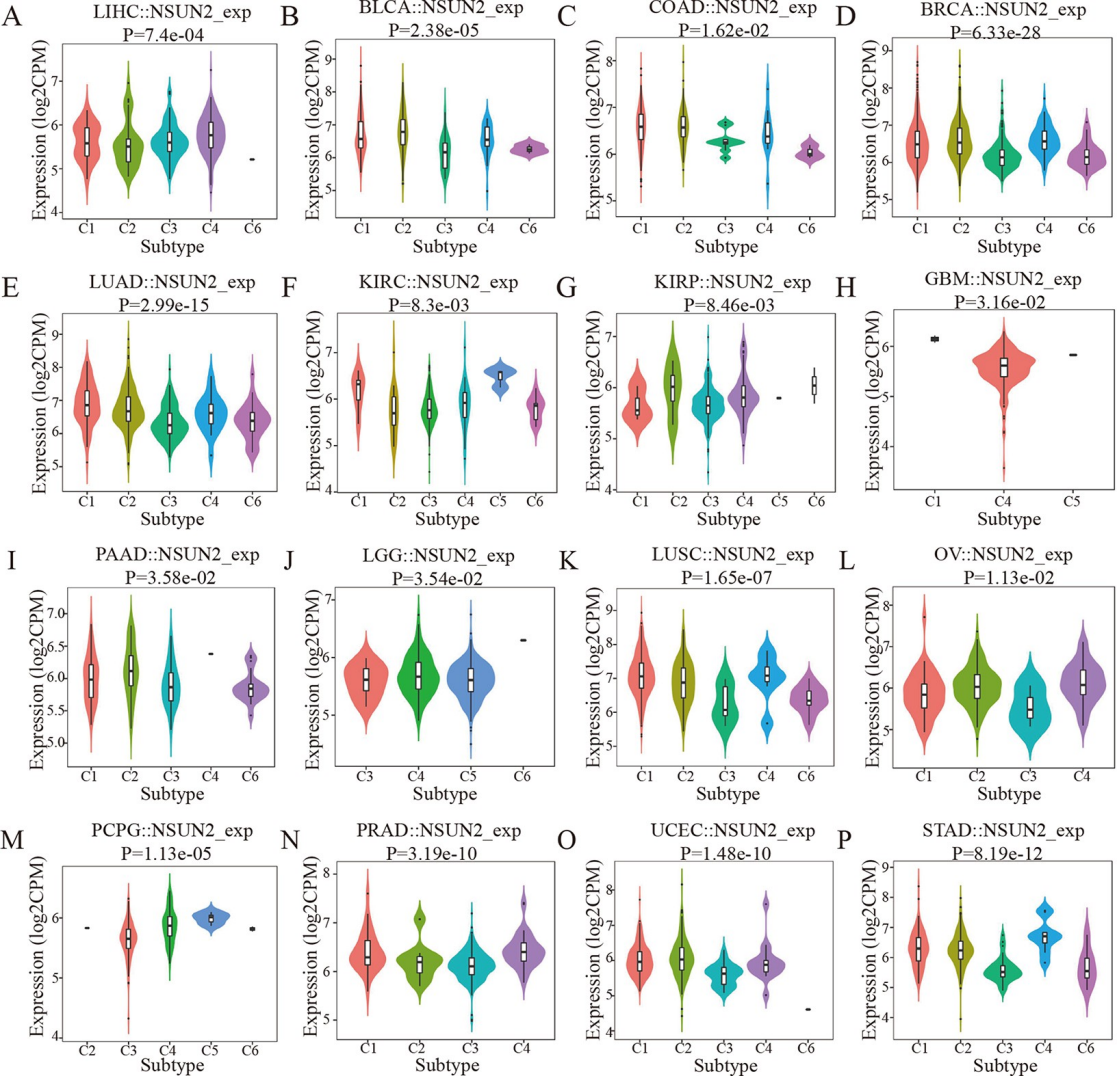
Correlation analysis between NSUN2 and immune subtypes in different cancer types. (A) In LIHC. (B) In BLCA. (C) In COAD. (D) In BRCA. (E) In LUAD. (F) In KIRC. (G) In KIRP. (H) In GBM. (I) In PAAD. (J) In LGG. (K) In LUSC. (L) In OV. (M) In PCPG. (N) In PRAD. (O) In UCEC. (P) In STAD. C1, wound healing. C2, IFN-γ dominant. C3, inflammatory. C4, lymphocyte depleted. C5, immunologically quiet. C6, TGF-β dominant.

Therefore, we further explored the relationships between the expression of NSUN2 and immune and molecular subtypes in different tumor types (S1 Table). The results showed that NSUN2 was differentially expressed in six immune subtypes (C1, C2, C3, C4, C5, C6) of the same tumor. For example, NSUN2 was highly expressed in the C4 and C1 subtypes in LIHC, expressed at relatively low levels in C2 and expressed at the lowest level in C6 (Fig 4A). In addition, the expression of NSUN2 was highly associated with different immune subtypes, such as LIHC and LUAD tumor types (Fig 4A–4P). Furthermore, we found that the expression of NSUN2 was greatly correlated with different molecular subtypes, such as HNSC and LIHC (S5 Fig). For example, there are four molecular subtypes of HNSC (mesenchymal, classical, basal and atypical), and NSUN2 was relatively highly expressed in the basal subtype (S5B Fig). All of the above findings indicated that NSUN2 is related to the immune and molecular subtypes of tumors.

### NSUN2 is related to immune cell infiltration, immune checkpoints (ICPs) and immunoregulatory genes

**Fig 5:**
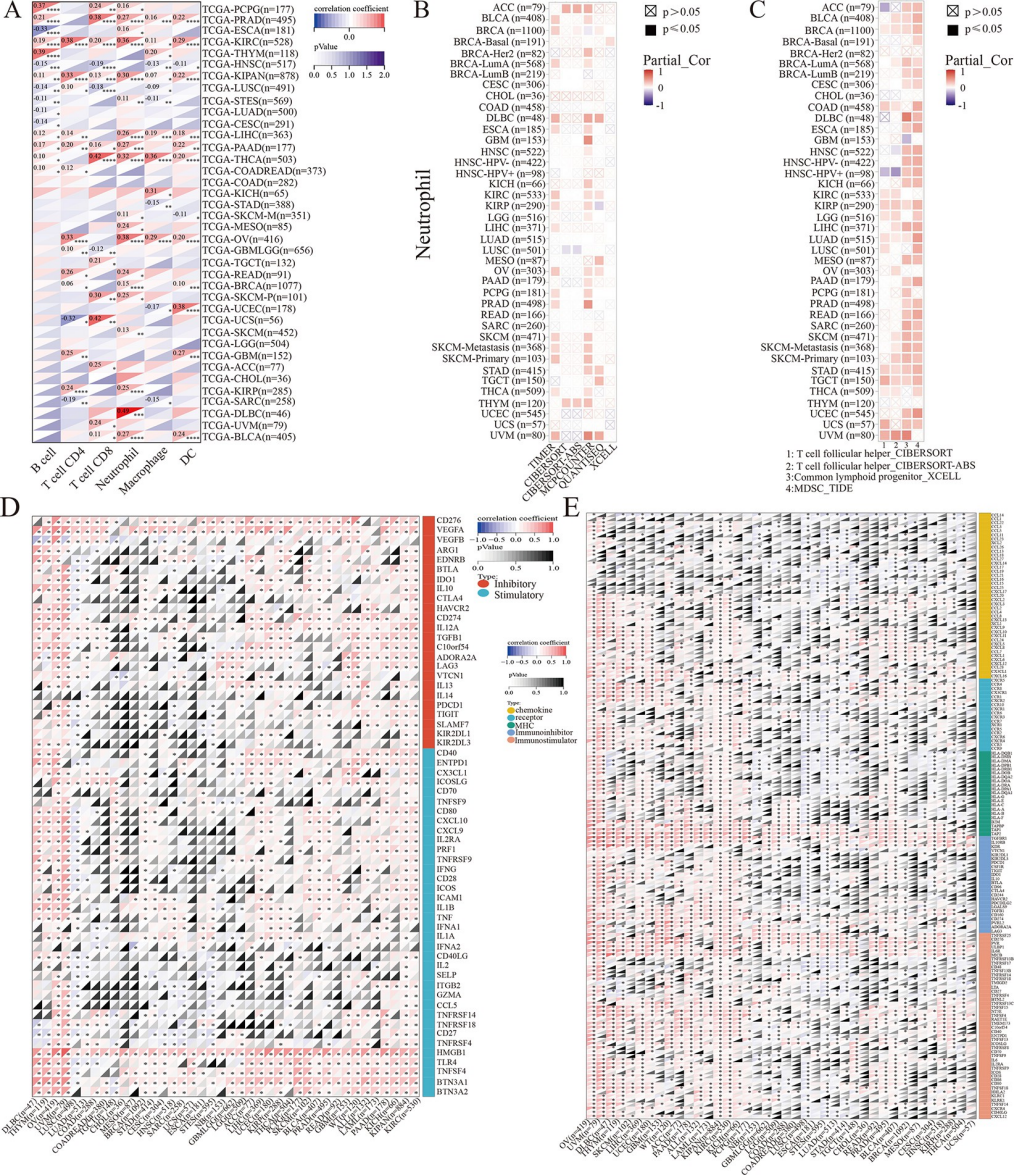
The relationships between the expression levels of NSUN2 and TILs or immune-related gene. (A) Expression analysis between NSUN2 and TILs including B cells, CD4+ T cells, CD8+ T cells, neutrophils, macrophages and dendritic cells. (B) The correlation between the expression levels of NSUN2 and neutrophils. (C) The relationships between the expression levels of NSUN2 and follicular helper T cells, common lymphoid progenitors and MDSCs. (D) The correlation between the expression levels of NSUN2 and immune checkpoint genes. (E) The correlation between the expression levels of NSUN2 and immune-regulated genes (*P<0.05, **P<0.01, ***P<0.001, ****P<0.0001).

In recent years, an increasing number of studies have shown that the tumor microenvironment (TME) is crucial in tumorigenesis and progression, and tumor-infiltrating lymphocytes (TILs) play a significant role in the TME [29, 30]. Therefore, the correlations between NSUN2 and immune cell infiltration, immune checkpoint genes and immunoregulatory genes were further analyzed. We found that the levels of infiltrating immune cells in 35 tumor types were significantly associated with NSUN2 expression, and the expression of NSUN2 was highly related to B cells, macrophages, CD4 T cells, neutrophils, CD8 T cells and dendritic cells (DCs) and most related to neutrophils. For example, the levels of these immune cells were significantly related to the expression of NSUN2 in KIRC, HNSC and LIHC (Fig 5A). NSUN2 expression was positively related to the levels of neutrophils in different tumor types (except LUSC), according to the TIMER, XCELL, CIBERSORT, QUANTISEQ, CIBERSORT-ABS and MCPCOUNTER algorithms (Fig 5B). Moreover, the levels of common lymphoid progenitor and myeloid-derived suppressor cells (MDSCs) in KIRP, STAD, LIHC, and other tumors were positively correlated with NSUN2 expression according to XCELL analysis and the TIDE algorithm, while NSUN2 expression was negatively correlated with the levels of T follicular helper cells in HNSC (Fig 5C).

Owing to the significance of ICPs in immune therapies, we analyzed the relationship between NSUN2 and ICP genes and immunoregulatory genes via the Sangerbox platform and found that the expression of NSUN2 was positively correlated with the expression ICP genes in multiple tumor types, such as CD276, VEGFA, and CD274. For example, the expression of NSUN2 was positively related to that of ICP genes in LIHC, OV, KIRC and BRCA, but negatively associated with that of ICPs genes in a few tumor types, such as LUSC (Fig 5D). In addition, the levels of some immunostimulators, immunoinhibitors, MHC molecules, chemokines and receptors were positively associated with NSUN2 expression levels in most tumor types, such as LIHC, KIRC and BRCA, while the expression of immune checkpoint-regulated genes was negatively correlated with the expression of NSUN2 in LUSC (Fig 5E). These results demonstrated that NSUN2 expression is closely related to tumor immunity and could serve as a potential target of immunotherapy.

### NSUN2-related proteins and genes

**Fig 6:**
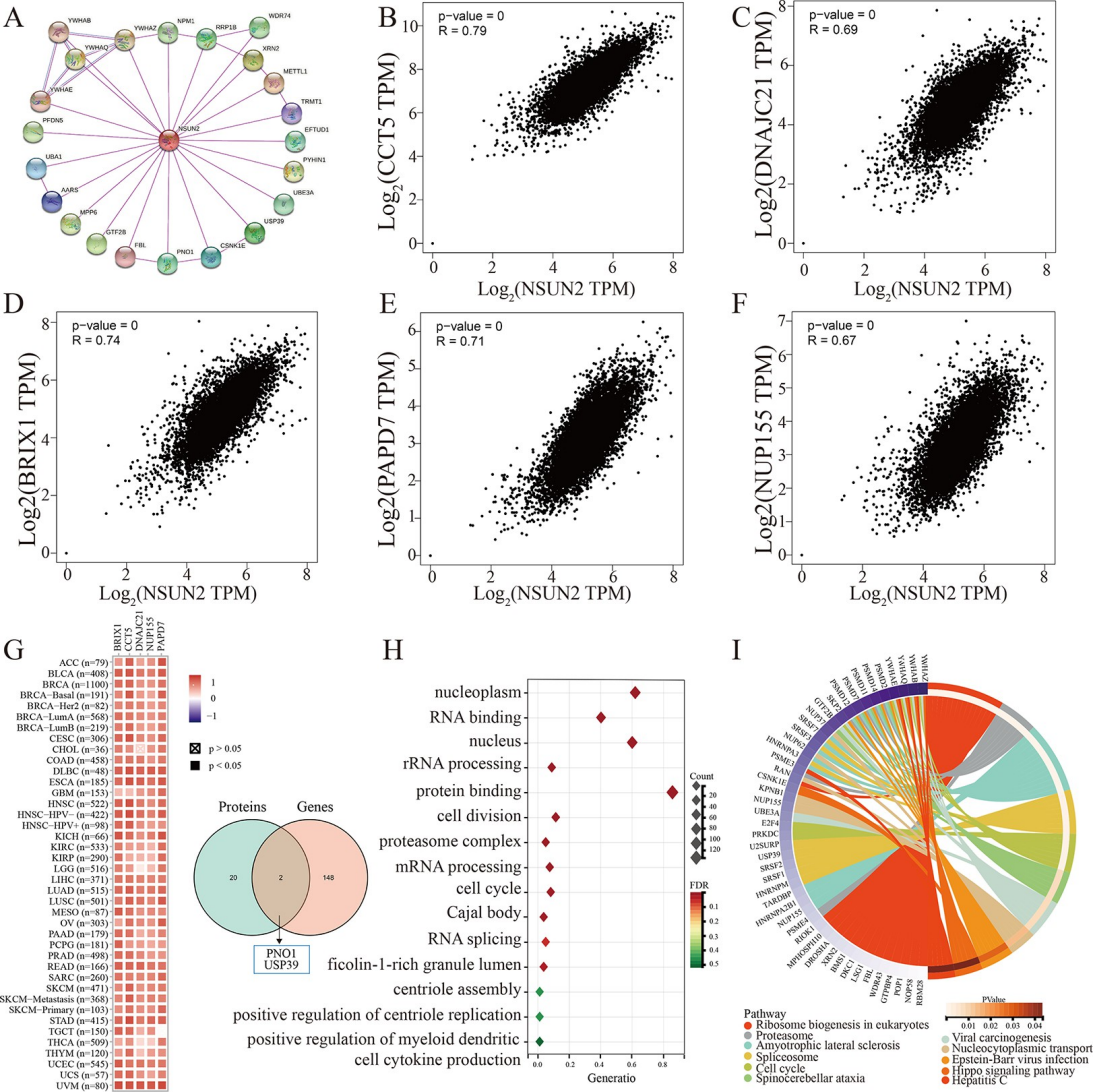
Enrichment analysis of NSUN2-related proteins and genes. (A) The proteins most closely related to NSUN2. (B-F) Analysis of NSUN2-related genes, including CCT5, DNAJC21, BRIX1, PAPD7 and NUP155. (G) The heatmap describes the expression of five NSUN2-correlated genes in different cancer types and the intersection of proteins and genes related to NSUN2. (H) GO analysis of NSUN2-related genes. (I) KEGG pathway analysis of NSUN2-related genes.

To further understand the interaction mechanism of NSUN2 in tumor progression, NSUN2-related proteins and genes were analyzed, and 22 NSUN2-related proteins were obtained from the STRING website after screening (only chose the “experiment”) (Fig 6A). In addition, the top 150 related genes of NSUN2 were obtained using the GEPIA website. We found that among 33 different tumor types, NSUN2 was positively correlated with chaperonin containing TCP1 subunit 5 (CCT5), DnaJ heat shock protein family (Hsp40) member C21 (DNAJC21), biogenesis of ribosomes BRX1 (BRIX1), terminal nucleotidyltransferase 4A (PAPD7) and Nucleoporin 155 (NUP155) genes (Fig 6B–6F). Moreover, PNO1 and USP39 overlapped between the related proteins and genes of NSUN2 in two datasets (Fig 6G).

We further combined NSUN2-related proteins and genes for gene ontology (GO) and KEGG pathway enrichment analysis. The results of GO analysis showed that protein binding, nucleoplasm, ficolin-1-rich granule lumen and positive regulation of myeloid dendritic cell cytokine production might play a crucial role in the effect of NSUN2 on tumors (Fig 6H). KEGG analysis further showed that NSUN2-related genes were enriched in ribosome biogenesis in eukaryotes, viral carcinogenesis, Epstein‒Barr virus infection, the Hippo signaling pathway and other pathways (Fig 6I).

### Coexpression networks of NSUN2 in LIHC, LUAD and HNSC

**Fig 7:**
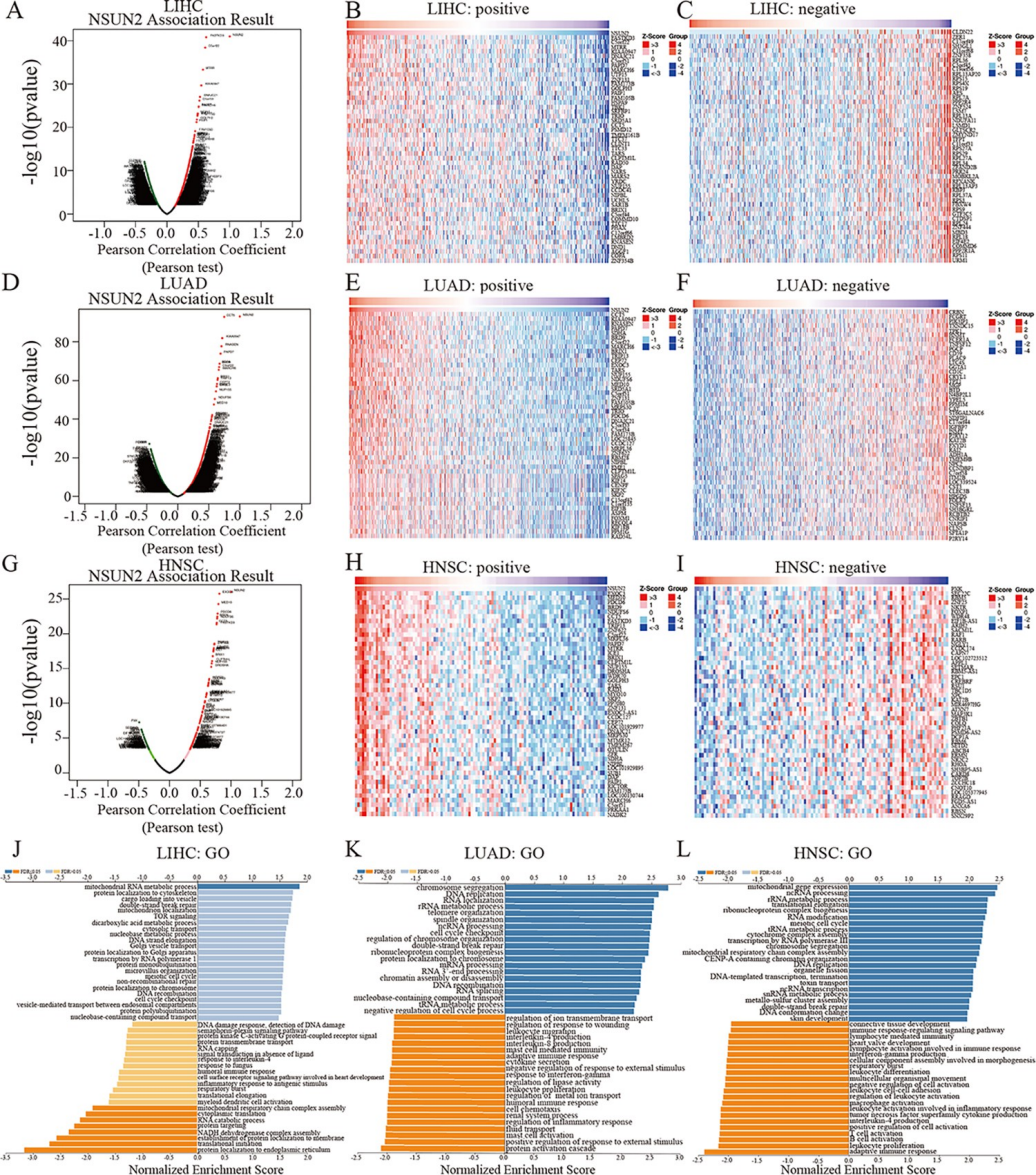
Coexpression genes analysis of NSUN2 in LIHC, LUAD and HNSC based on the LinkedOmics website. (A) Genes related to NSUN2 based on the Pearson test in LIHC cohorts. (B) The top 50 positively related genes of NSUN2 in LIHC. (C) The top 50 negatively related genes of NSUN2 in LIHC. (D) Genes related to NSUN2 in LUAD cohorts. (E) The top positively related genes of NSUN2 in LUAD. (F) The top 50 genes negatively related to NSUN2 in LUAD. (G) Genes related to NSUN2 in HNSC cohorts. (H) The top 50 genes positively related to NSUN2 in HNSC. (I) The top 50 genes negatively related to NSUN2 in HNSC. (J) Biological process analysis of NSUN2 in LIHC cohorts. (K) Biological process analysis of NSUN2 in LUAD cohorts. (L) Biological process analysis of NSUN2 in HNSC.

The coexpression networks of NSUN2 were analyzed in LIHC, LUAD and HNSC through the LinkedOmics web portal, and positively correlated genes and negatively correlated genes related to NSUN2 were obtained (Fig 7A–7I). Taking HNSC as an example, 187 genes were found to be positively related to NSUN2, while 88 genes were negatively related to NSUN2 (Fig 7G). In addition, the 50 genes most closely related to NSUN2 in LIHC, LUAD and HNSC were obtained (Fig 7B, 7C, 7E, 7F, 7H, 7I). Subsequently, the biological process categories of GO and KEGG pathways of NSUN2-related genes were analyzed by gene set enrichment analysis (GSEA) (Fig 7J–7L, S6A–S6C Fig). We found that NSUN2 was associated with the humoral immune response in LIHC and with the adaptive immune response, humoral immune response and other biological processes in LUAD, while in HNSC, NSUN2 was significantly correlated with T-cell activation, the immune response-regulating signaling pathway and B-cell activation (Fig 7J–7L). Furthermore, the relationships between NSUN2 and immune cells in three tumor types were further analyzed by the TIMER database, and we found that NSUN2 was associated with the infiltration levels of immune cells such as DCs, neutrophils and CD8 T cells (S6D Fig). The results revealed that NSUN2 might have an important function in tumor progression by participating in the immune response.

### The subcellular localization and function of NSUN2 in LIHC, LUAD and HNSC

**Fig 8:**
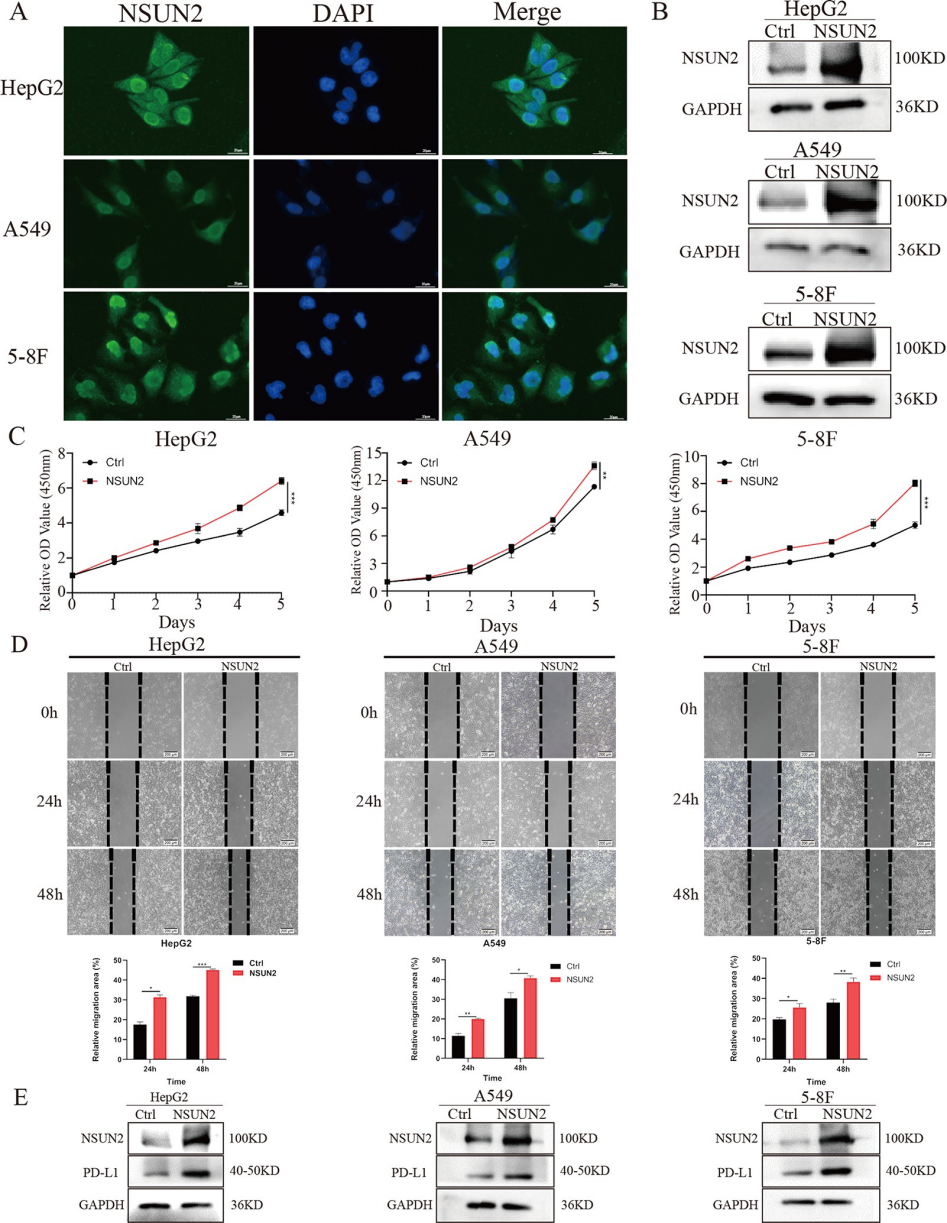
The subcellular localization and functions of NSUN2 in LIHC, LUAD and HNSC. (A) The subcellular localization of NSUN2 in HepG2, A549 and 5-8F cells. (B) Detection of the overexpression efficiency of NSUN2 in cell lines. (C) The effect of NSUN2 overexpression on cell proliferation was tested by CCK-8 assay in HepG2, A549 and 5-8F cell lines. (D) The effect of NSUN2 overexpression on cell migration was tested by wound healing assay in HepG2, A549 and 5-8F cell lines. (E) The effect of NSUN2 overexpression on the expression of PD-L1 in HepG2, A549 and 5-8F cell lines.

The subcellular localization of NSUN2 was first explored with the GeneCards web tool, and we found that NSUN2 was mainly located in the nucleus (S6E Fig). Furthermore, HepG2, A549 and 5-8F cell lines were selected as representative cell lines of LIHC, LUAD and HNSC and were used to investigate the subcellular location and function of NSUN2 in tumors. The results showed that NSUN2 was widely located in the nucleus and cytoplasm but is mainly expressed in the nucleus (Fig 8A), which is similar to the bioinformatic prediction. Moreover, we overexpressed NSUN2 in these three cell lines, and the western blotting results verified the overexpression efficiency of NSUN2 (Fig 8B), Then, CCK-8 and wound healing assays demonstrated that NSUN2 could promote cell proliferation and migration (Fig 8C, 8D). In addition, because the checkpoint PD-L1 plays an important role in tumor escape, we further overexpressed NSUN2 and found that NSUN2 increased the protein expression of PD-L1 (Fig 8E). These results reveal that NSUN2 can serve as an oncogene in tumors and is involved in tumor progression and the immune response.

## Discussion

Changes in epigenetic modification are a basic feature of tumors [31]. In recent years, an increasing number of researchers have focused on the relationship between alterations in RNA modifications and tumorigenesis, including the study of m6A, m1A and m5C modifications [32–35]. M5C, a kind of posttranscriptional modification of RNA, is essential in the malignant progression of cancer [36, 37]. Many researchers have demonstrated that m5C is related to the tumor immune microenvironment. For example, Pan et al. found that 14 lncRNAs related to m5C are associated with the immune microenvironment in a study of lung adenocarcinoma [38]. Among the regulators of m5C modification, NSUN2 is an important methyltransferase; however, NSUN2 is less studied in the context of tumor, and its functions in immunology are largely unclear.

In this study, the expression of NSUN2 in different cancer types was first analyzed with the TIMER database and UCSC website, and we found that NSUN2 was highly expressed in most types of tumors compared with normal tissues. Similar to previous studies, NSUN2 was indeed highly expressed in gastric cancer, ESCC and nasopharyngeal carcinoma [14, 15, 39], which indicated that NSUN2 might be highly expressed in other tumor types and can be used as a potential pan-cancer biomarker. At the same time, some researchers have shown that NSUN2 plays critical roles in tumors [40]. For example, Xu et al. found that NSUN2 can methylate the mRNA of autotaxin and increase the protein expression of autotaxin in glioma, thus promoting the migration of glioma cells [41]. In uveal melanoma, NSUN2 participates in the m5C modification process and promotes the migration of uveal melanoma cells [42]. Moreover, Tong et al. also demonstrated that NSUN2 exerted critical roles in the proliferation of nasopharyngeal carcinoma cells [39]. These studies have shown that NSUN2 plays an oncogenic role and promotes tumor development and progression. In our study, NSUN2 was also significantly overexpressed in other tumor types. Therefore, NSUN2 may play oncogenic roles in multiple tumor types. In addition, high NSUN2 expression was associated with a poor prognosis in LIHC, KIRC, LUAD and other cancer types. Studies have confirmed that high NSUN2 expression is a poor prognostic factor in GC and HNSC [14, 43], which highlights that NSUN2 serves as a poor prognostic factor and can be used as a pan-cancer biomarker.

Among cancer-associated fibroblasts, stromal cells, immune cells and other cells in the tumor microenvironment (TME), immune cells exert crucial roles in the supervision of tumor cell escape [44]. In our study, we found that NSUN2 was relatively highly expressed in immune cells using the TISCH web tool. To describe the functions of NSUN2 in immunity, the correlation between NSUN2 and immune subtypes and molecular subtypes was investigated with the TISIDB database, and we found that NSUN2 was significantly correlated with immune subtypes and molecular subtypes in most cancer types, which indicated that NSUN2 is not only a poor prognosis biomarker in pan-cancer but also participates in immunology. Some researchers have demonstrated that NSUN2 can engage in immunity. NSUN2 can promote the translation of IL-17A in T cells by methylating interleukin-17A (IL-17A) [45]. Furthermore, correlation analysis of immunology showed that NSUN2 expression was related to the levels of infiltrating immune cells, such as neutrophils, CD4 T cells, B cells, macrophages, CD8 T cells and dendritic cells, and was most closely related to the levels of neutrophils. Analysis of the TIMER database also proved that NSUN2 was significantly related to the levels of neutrophils and myeloid-derived suppressor cells (MDSCs). Neutrophils are markers of chronic inflammation, and long-standing inflammation can promote the occurrence of tumors. In addition, MDSCs can inhibit adaptive immunity in the tumor microenvironment to promote the occurrence of tumors [46]. Some studies have explored the role of NSUN2 in immunology. Miao et al. found that a lack of NSUN2 can suppress the infiltration of T cells in the context of abdominal aortic aneurysm (AAA), while the presence of NSUN2 can mediate the occurrence of AAA by promoting the migration of T cells [47]. NSUN2 can also regulate immune infiltration in nasopharyngeal carcinoma [39], which reveals that NSUN2 may act on immune cells to play a role in tumor progression. In addition, NSUN2 expression was also found to be significantly correlated with the expression of immune checkpoint genes and immunoregulatory genes. Recently, PD-1/PD-L1 antibodies applied to block PD-1 signaling or other immune signaling pathways have achieved great therapeutic effects in tumors [48]. These results suggested that NSUN2 plays a crucial role in immunity by regulating the infiltration of immune cells and the expression of immune-related molecules; thus, NSUN2 is expected to become a target gene of antitumor drugs in immune therapies. Moreover, in the coexpression network analysis of NSUN2 in LIHC, LUAD and HNSC, we showed that NSUN2 indeed participates in the regulation of immunity in cancers, such as LUAD and HNSC, and that NSUN2 participates in the adaptive immune response, interleukin-4 production and other immune-related responses. This revealed that targeting NSUN2 has great potential in the context of immunotherapy.

We found that NSUN2 was widely expressed in the nucleus and cytoplasm but was mainly expressed in the nucleus, which is similar to the prediction of subcellular location and reflects that NSUN2 is highly expressed in tumors to a large extent. In addition, CCK-8 assays and wound healing assays also demonstrated that NSUN2 can promote cell proliferation and migration and is related to the expression of PD-L1, which reveals that NSUN2, as an m5C methyltransferase, might regulate the modification of downstream target genes, thus participating in immune regulation and promoting tumorigenesis as an oncogene. NSUN2 expression was analyzed through different databases, and there are some differences in data derived from different databases. Therefore, in future work, more experiments need to be performed to verify the expression profiles and functions of NSUN2 in tumor progression and immune therapy.

## Conclusion

In summary, NSUN2 was highly expressed in most tumor types and correlated with poor prognosis and immune infiltration. Moreover, the subcellular location and function of NSUN2 in cell proliferation and migration were verified, suggesting that NSUN2 is mainly located in the nucleus and that highly expressed-NSUN2 can serve as an oncogene as well as a prognostic biomarker across cancers, suggesting that targeting NSUN2 might be a potential therapeutic strategy for tumor immunotherapy.

## Supporting information

S1 Fig Expression of NSUN2 in tumor types. (A) Analysis of mRNA expression of NSUN2 in different cancer types by TIMER database. (B) Protein expression of NSUN2 in glioblastoma multiformine. (C) Protein expression of NSUN2 in clear cell RCC (*P<0.05, **P<0.01, ***P<0.001).
(TIF)

S2 Fig Analysis of the individual cancer stages of NSUN2 in different cancer types by UALCAN database. (A) In LIHC. (B) In HNSC. (C) In BRCA. (D) In CESC. (E) In CHOL. (F) In COAD. (G) In ESCA. (H) In BLCA. (I) In KIRC. (J) In KIRP. (K) In LUAD. (L) In LUSC. (M) In KICH. (N) In READ. (O) In STAD. (P) In UCEC (*P<0.05, **P<0.01, ***P<0.001).
(TIF)

S3 Fig Survival curve of NSUN2 by the Kaplan‒Meier plotter database and PrognoScan database. (A-B) RFS in READ and SARC cohorts. (C-E) OS in BLCA, PCPG and EACA cohorts. (F) OS in LUAD cohorts. (G-I) OS, RFS and DSS in BRCA cohorts. (J) OS in OV cohorts. (K) DFS in CRC cohorts. OS, overall survival. RFS, relapse-free survival. DSS, disease Specific survival. DFS, disease free survival.
(TIF)

S4 Fig RNA modification and expression of NSUN2 in immune cells. (A) Analysis between NSUN2 and RNA modification-related genes. (B) Protein expression of NSUN2 in different seven cells or tissue types. (C) Expression of NSUN2 in immune cells.
(TIF)

S5 Fig Correlation analysis between NSUN2 and molecular subtype in different cancer types. (A) In LIHC. (B) In HNSC. (C) In BRCA. (D) In ESCA. (E) In OV. (F) In LGG. (G) In ACC. (H) In PCPG. (I) In KIRP. (J) In UCEC. (K) In STAD. (L) In PRAD.
(TIF)

S6 Fig Coexpression analysis of NSUN2-related genes in LIHC, LUAD and HNSC, and analysis between NSUN2 expression and the levels of immune cells. (A-C) KEGG pathway analysis of NSUN2-related gene in LIHC, LUAD and HNSC cohorts. (D) Expression analysis between NSUN2 and TILs including B cells, CD8+ T cells, CD4+ T cells, macrophages, neutrophils and dendritic cells based on the TIMER database. (E) The subcellular localization prediction of NSUN2.
(TIF)

S1 Table Sample number of immune and molecular subtypes. (DOCX)

S1 Raw images Original western blot. (TIF)
