# Supplementary figures and images for: NOP2/Sun RNA methyltransferase 2 is a potential pan-cancer prognostic biomarker and is related to immunity
Source: PLoS One. 2023 Sep 28;18(9):e0292212. doi: 10.1371/journal.pone.0292212 (PMC10538670; doi:10.1371/journal.pone.0292212)

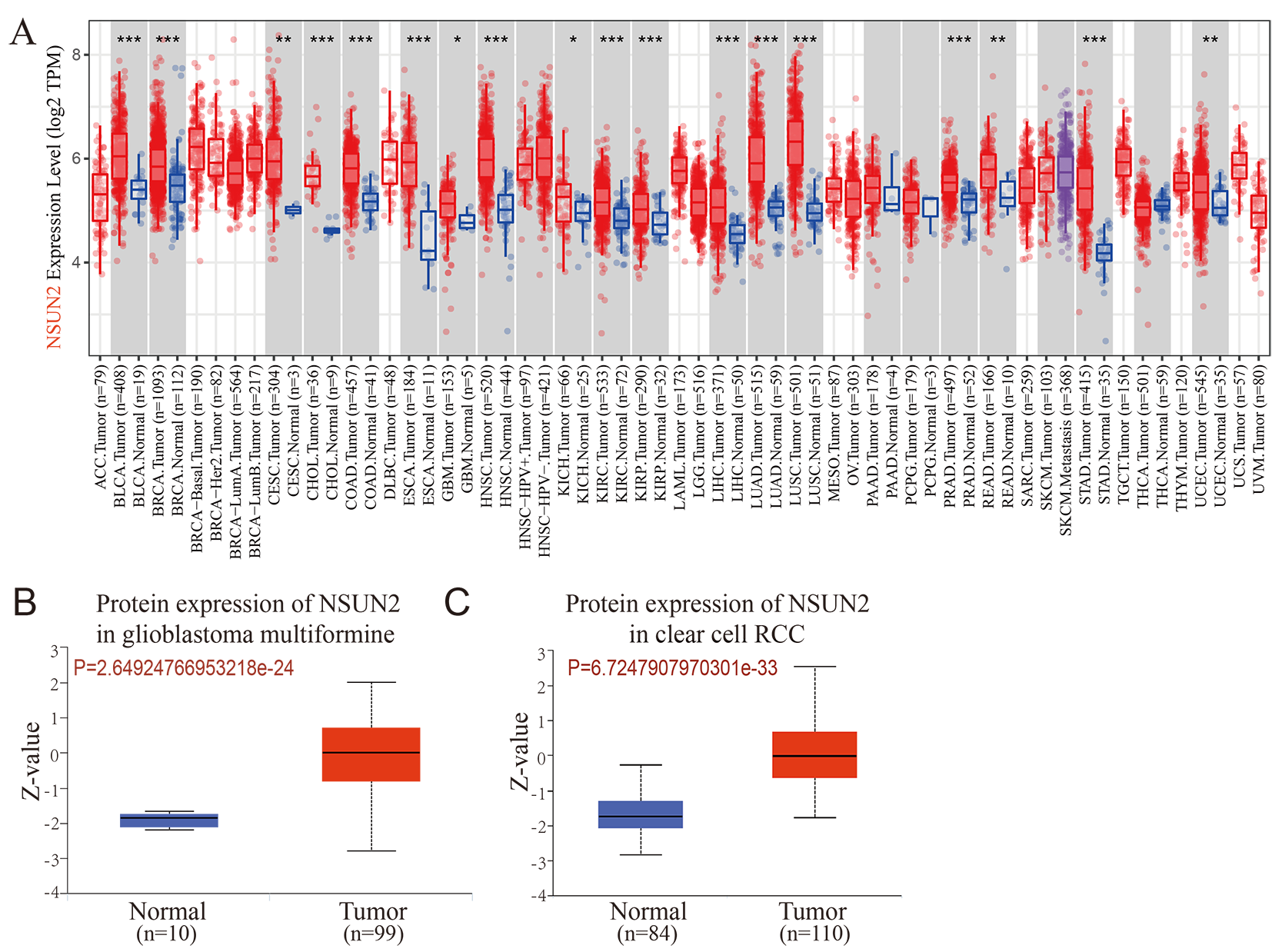

Supplement: S1 Fig — (A) Analysis of mRNA expression of NSUN2 in different cancer types by TIMER database. (B) Protein expression of NSUN2 in glioblastoma multiformine. (C) Protein expression of NSUN2 in clear cell RCC (*P<0.05, **P<0.01, ***P<0.001). (TIF) [file pone.0292212.s001.tif]

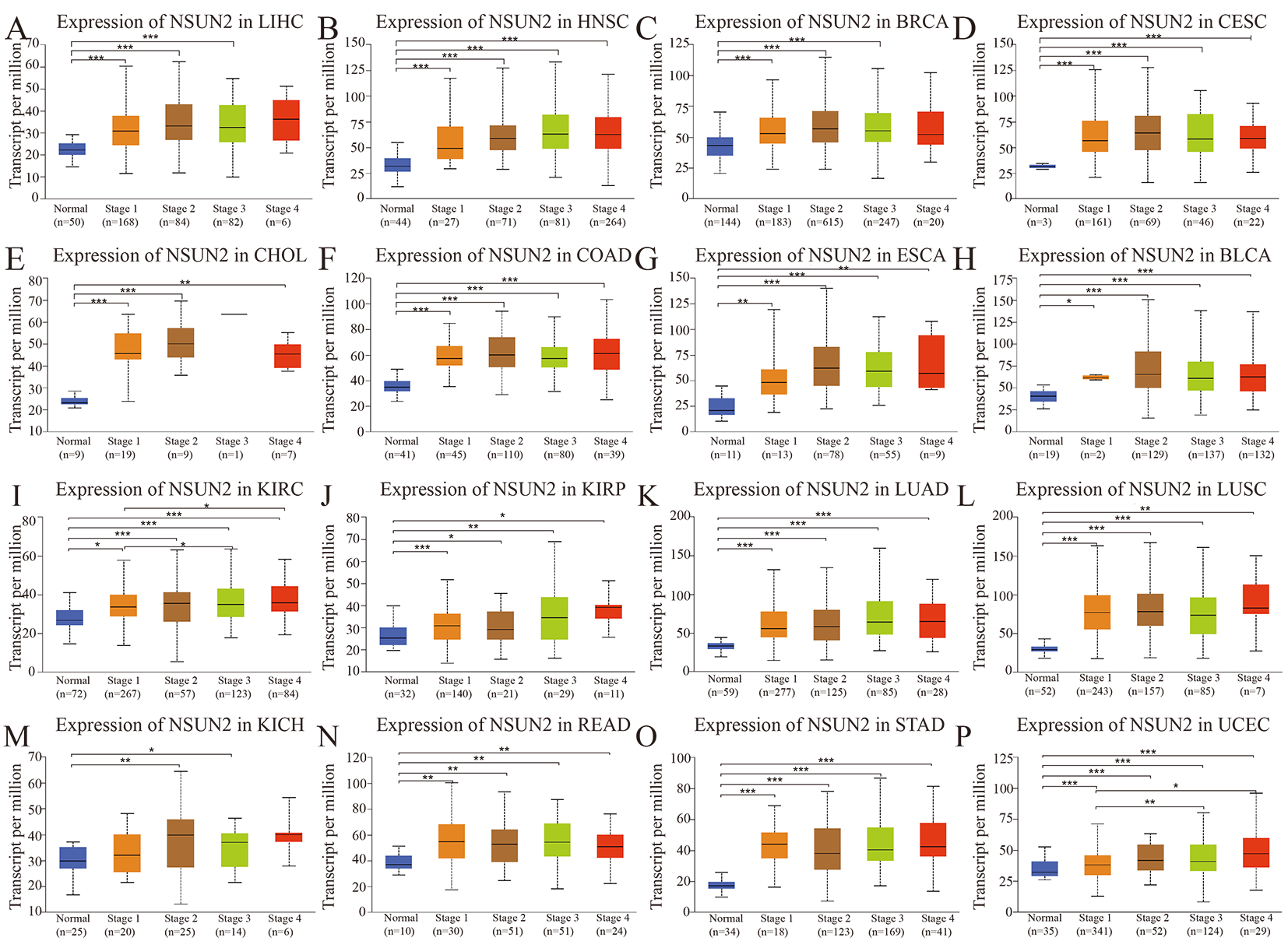

Supplement: S2 Fig — (A) In LIHC. (B) In HNSC. (C) In BRCA. (D) In CESC. (E) In CHOL. (F) In COAD. (G) In ESCA. (H) In BLCA. (I) In KIRC. (J) In KIRP. (K) In LUAD. (L) In LUSC. (M) In KICH. (N) In READ. (O) In STAD. (P) In UCEC (*P<0.05, **P<0.01, ***P<0.001). (TIF) [file pone.0292212.s002.tif]

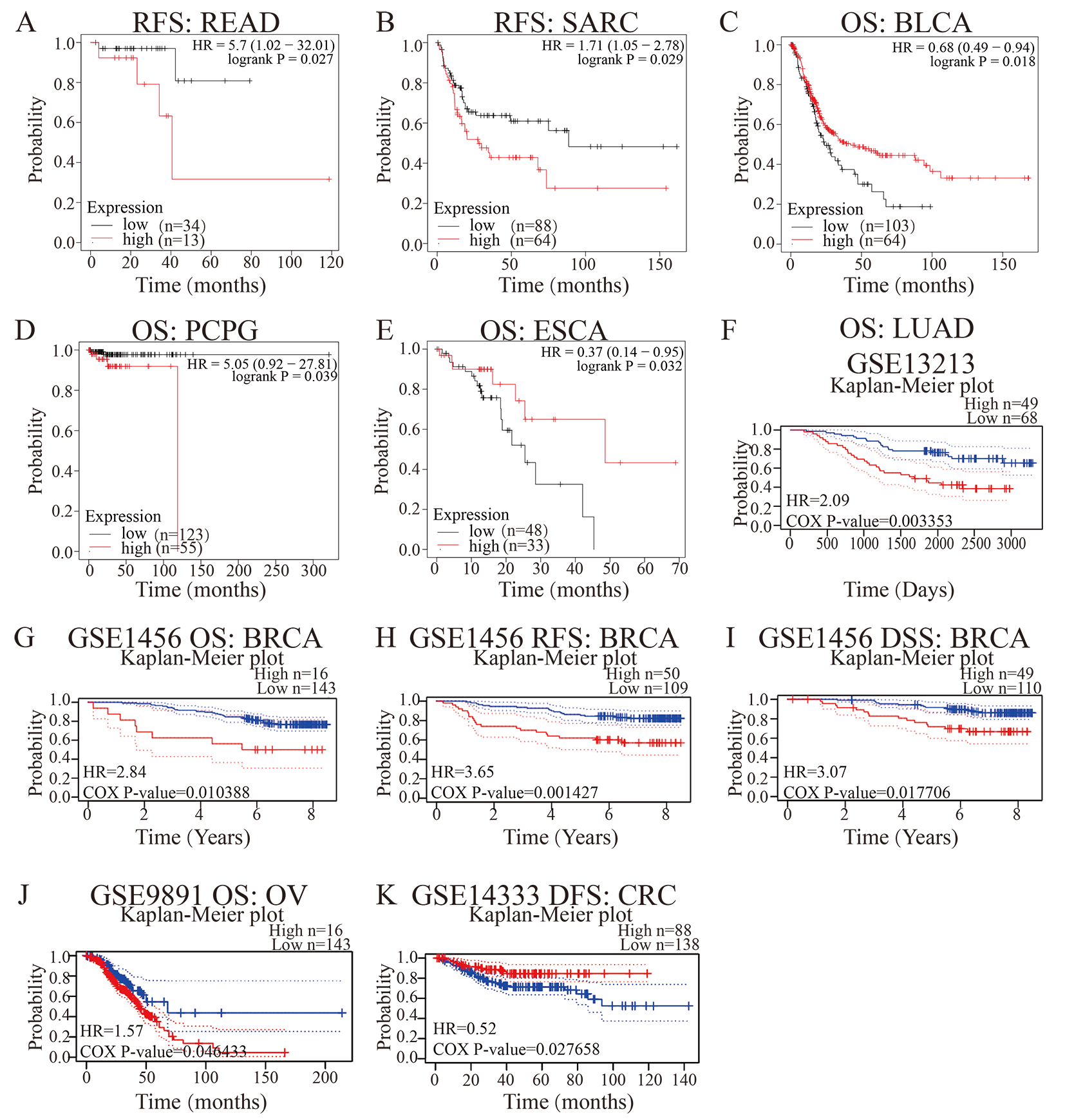

Supplement: S3 Fig — (A-B) RFS in READ and SARC cohorts. (C-E) OS in BLCA, PCPG and EACA cohorts. (F) OS in LUAD cohorts. (G-I) OS, RFS and DSS in BRCA cohorts. (J) OS in OV cohorts. (K) DFS in CRC cohorts. OS, overall survival. RFS, relapse-free survival. DSS, disease Specific survival. DFS, disease free survival. (TIF) [file pone.0292212.s003.tif]

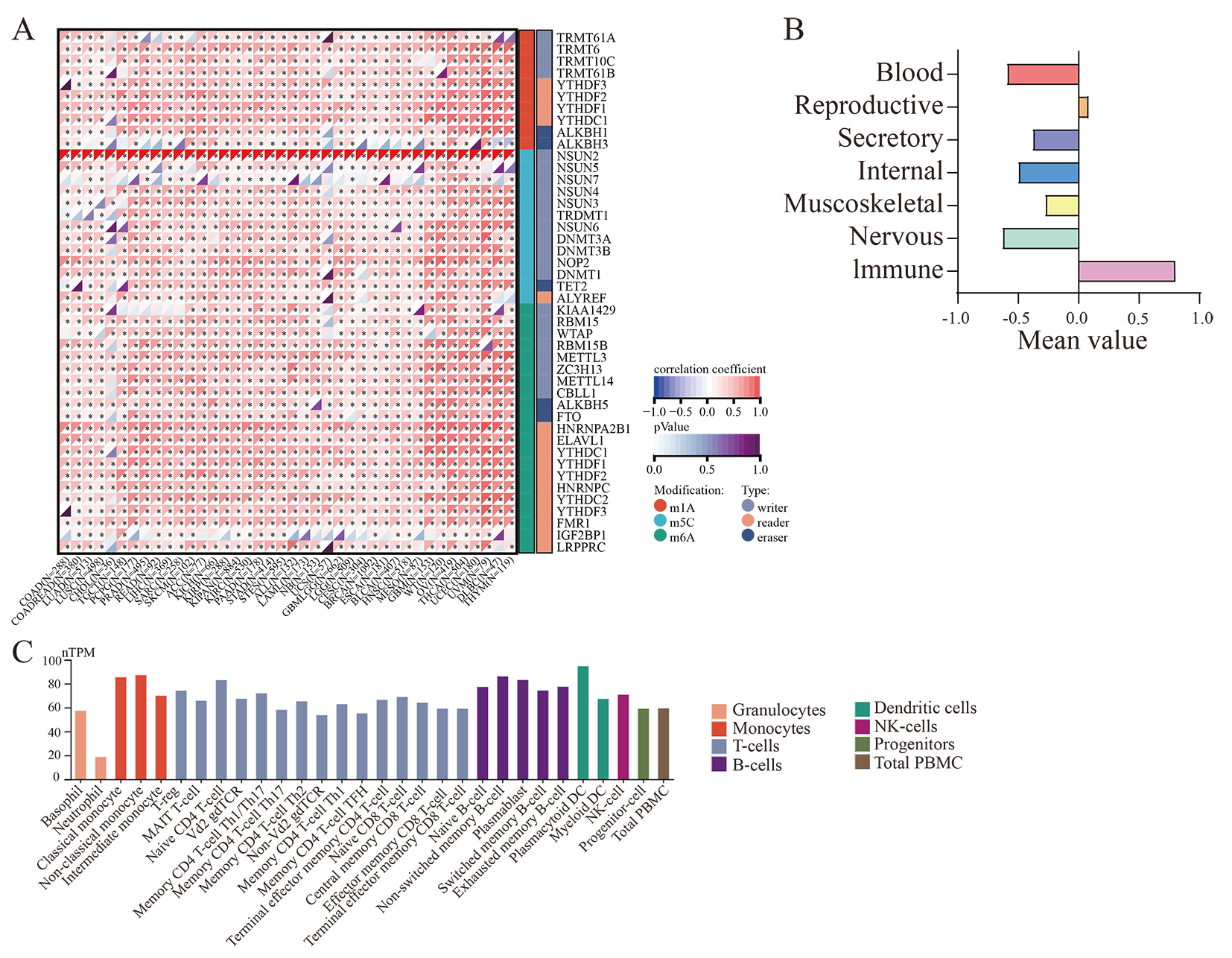

Supplement: S4 Fig — (A) Analysis between NSUN2 and RNA modification-related genes. (B) Protein expression of NSUN2 in different seven cells or tissue types. (C) Expression of NSUN2 in immune cells. (TIF) [file pone.0292212.s004.tif]

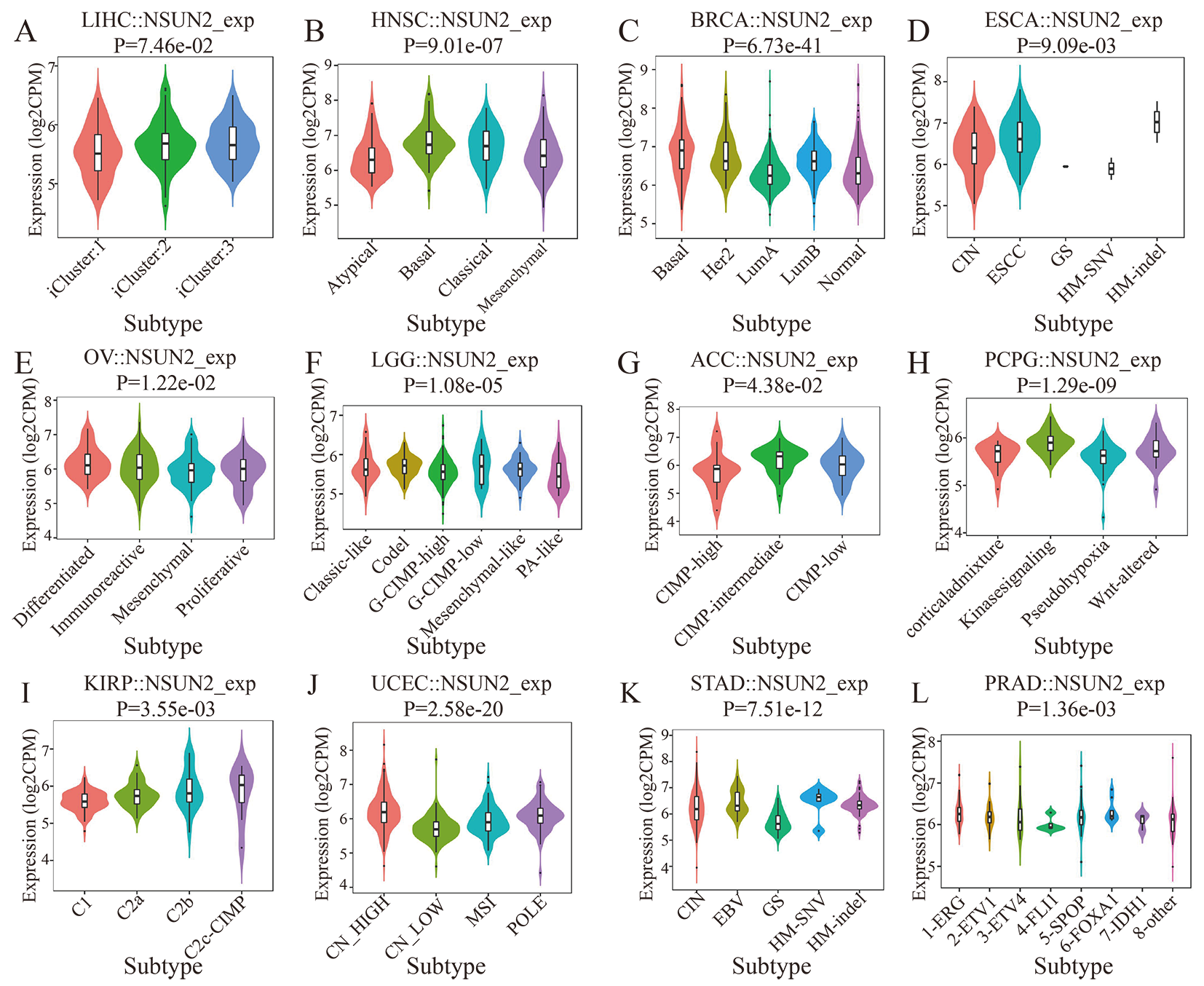

Supplement: S5 Fig — (A) In LIHC. (B) In HNSC. (C) In BRCA. (D) In ESCA. (E) In OV. (F) In LGG. (G) In ACC. (H) In PCPG. (I) In KIRP. (J) In UCEC. (K) In STAD. (L) In PRAD. (TIF) [file pone.0292212.s005.tif]

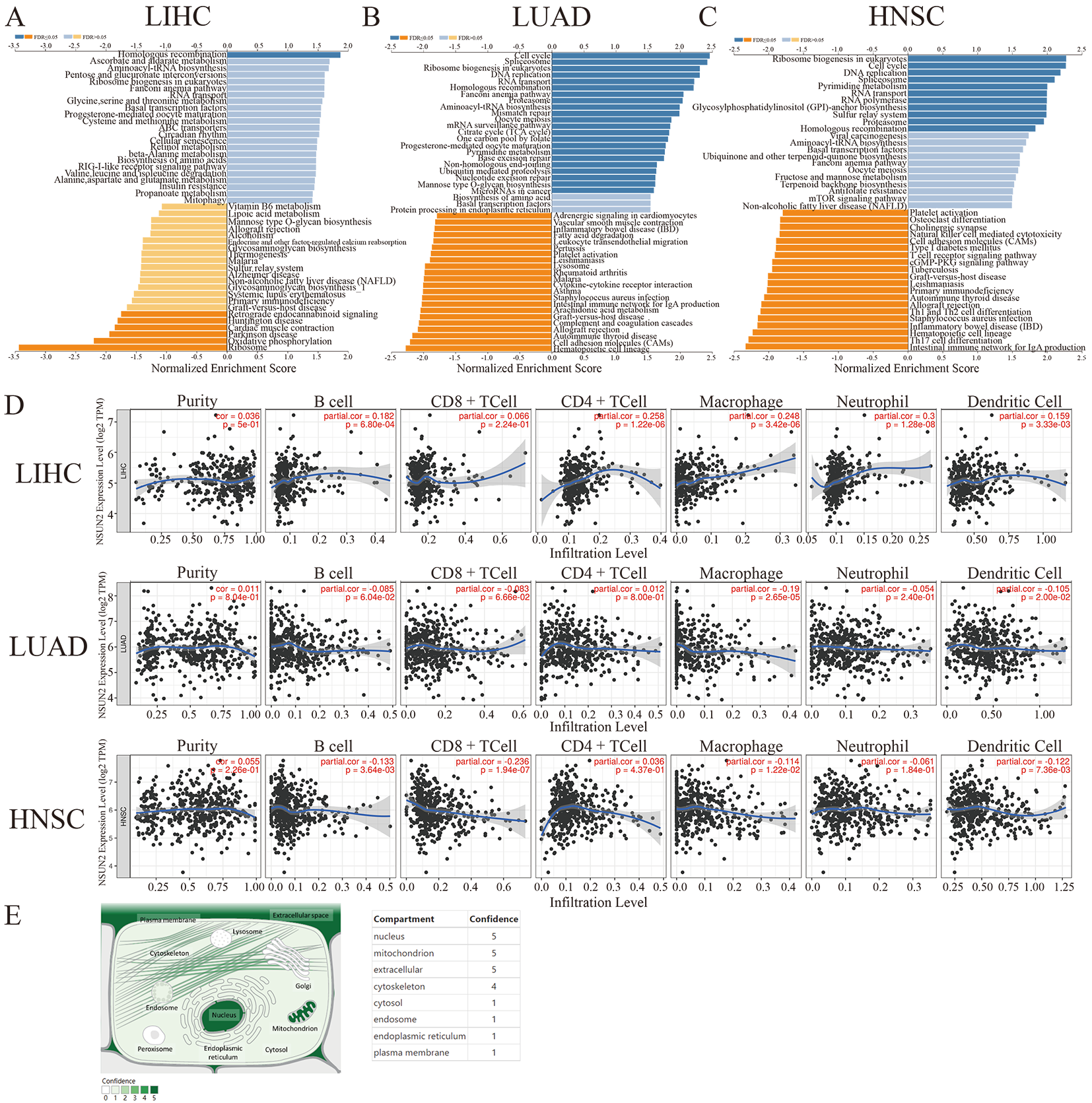

Supplement: S6 Fig — (A-C) KEGG pathway analysis of NSUN2-related gene in LIHC, LUAD and HNSC cohorts. (D) Expression analysis between NSUN2 and TILs including B cells, CD8+ T cells, CD4+ T cells, macrophages, neutrophils and dendritic cells based on the TIMER database. (E) The subcellular localization prediction of NSUN2. (TIF) [file pone.0292212.s006.tif]

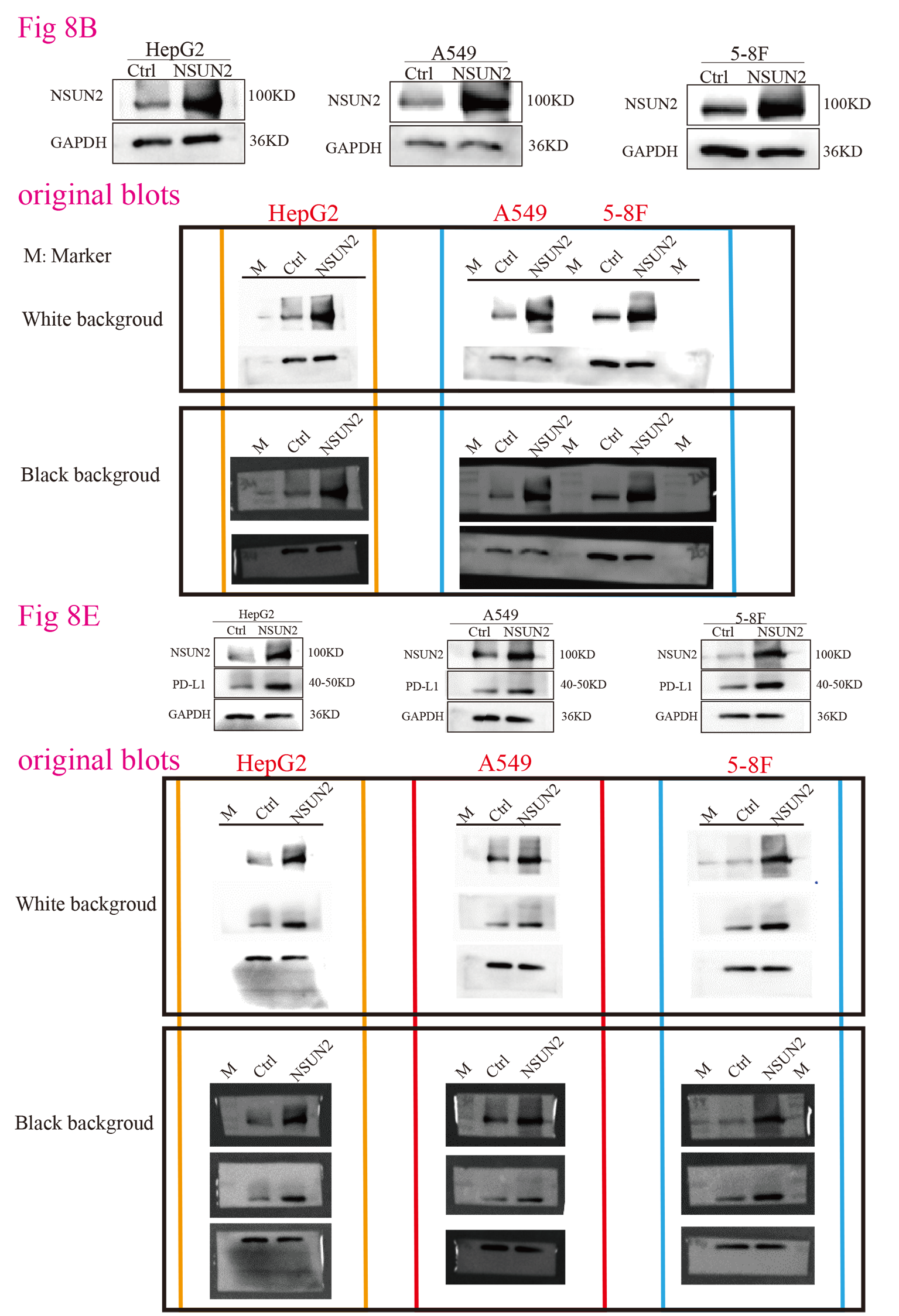

Supplement: S1 Raw images — (TIF) [file pone.0292212.s008.tif]
