# Supplementary material for: NOP2/Sun RNA methyltransferase 2 is a potential pan-cancer prognostic biomarker and is related to immunity
Source: PLoS One. 2023 Sep 28;18(9):e0292212. doi: 10.1371/journal.pone.0292212 (PMC10538670; doi:10.1371/journal.pone.0292212)
Supplement: S1 Table — (DOCX) [file pone.0292212.s007.docx]

**S1 Table. Sample number of immune and molecular subtypes.**

| **Cancer Abbreviation** | **TCGA Cancer Type** | **Total sample number of immune/ molecular subtypes** | **Sample Number of** **immune subtypes**  **(C1/C2/C3/C4/C5/C6)** | **Sample Number of molecular subtypes**  **(Left to Right)** |
| --- | --- | --- | --- | --- |
| ACC | Adrenocortical carcinoma | 78/78 | 1/1/23/49/3/1 | 19/27/32 |
| BLCA | Bladder Urothelial Carcinoma | 397/NA | 173/164/21/36/3 | NA |
| BRCA | Breast invasive carcinoma | 1082/1081 | 369/390/191/92/40 | 172/73/508/191/137 |
| CESC | Cervical squamous cell carcinoma and endocervical adenocarcinoma | 300/NA | 77/217/NA/6/NA/NA | NA |
| CHOL | Cholangiocarcinoma | 35/NA | 7/2/17/8/1 | NA |
| COAD | Colon adenocarcinoma | 441/NA | 332/85/9/12/3 | 226/49/6/60 |
| DLBC | Large B-cell Lymphoma | NA | NA | NA |
| ESCA | Esophageal carcinoma | NA/169 | NA | 74/90/1/2/2 |
| GBM | Glioblastoma multiforme | 153/119 | 2/NA/NA/150/1/NA | 47/2/5/12/53 |
| HNSC | Head and Neck squamous cell carcinoma | 514/276 | 128/379/2/2/3 | 67/87/48/74 |
| KICH | Kidney Chromophobe | 65/NA | 2/38/12/13/NA | NA |
| KIRC | Kidney renal clear cell carcinoma | 515/NA | 7/20/445/27/3/13 | NA |
| KIRP | Kidney renal papillary cell carcinoma | 279/161 | 3/4/202/66/2/2 | 95/35/22/9 |
| LGG | Brain Lower Grade Glioma | 514/511 | NA/NA/10/147/356/1 | 23/171/234/12/45/26 |
| LIHC | Liver hepatocellular carcinoma | 362/182 | 22/45/135/159/1 | 64/55/63 |
| LUAD | Lung adenocarcinoma | 457/NA | 83/147/179/20/28 | NA |
| LUSC | Lung squamous cell carcinoma | 486/170 | 275/182/8/7/14 | 42/63/26/39 |
| LAML | Acute Myeloid Leukemia | NA | NA | NA |
| MESO | Mesothelioma | 83/NA | 32/21/8/11/11 | NA |
| OV | Ovarian serous cystadenocarcinoma | 269/293 | 46/159/3/61/NA/NA | 66/78/71/78 |
| PAAD | Pancreatic adenocarcinoma | 151/NA | 57/32/40/1/21 | NA |
| PCPG | Pheochromocytoma and Paraganglioma | 178/173 | NA/1/107/63/5/2 | 22/68/61/22 |
| PRAD | Prostate adenocarcinoma | 405/333 | 35/18/307/45/NA/NA | 152/28/14/4/37/9/3/86 |
| READ | Rectum adenocarcinoma | 156/118 | 127/18/9/1/NA/1 | 102/9/4/3 |
| SARC | Sarcoma | 223/NA | 64/38/42/59/20 | NA |
| SKCM | Skin Cutaneous Melanoma | 103/315 | 41/27/14/19/NA/2 | 150/27/92/46 |
| STAD | Stomach adenocarcinoma | 391/383 | 129/210/36/9/NA/7 | 223/30/50/7/73 |
| TGCT | Testicular Germ Cell Tumors | 149/NA | 42/104/2/1/NA/NA | NA |
| THYM | Thymoma | NA | NA | NA |
| THCA | Thyroid carcinoma | 499/NA | 2/13/459/22/NA/3 | NA |
| UCS | Uterine Carcinosarcoma | 57/NA | 41/14/NA/2/NA/NA | NA |
| UCEC | Uterine Corpus Endometrial Carcinoma | 528/507 | 247/212/52/16/NA/1 | 160/144/124/79 |
| UVM | Uveal Melanoma | 80/NA | NA/NA/30/48/2/NA | NA |
